# Supplementary figures and images for: Genome-scale metabolic model of the diatom Thalassiosira pseudonana highlights the importance of nitrogen and sulfur metabolism in redox balance
Source: PLoS One. 2021 Mar 24;16(3):e0241960. doi: 10.1371/journal.pone.0241960 (PMC7990286; doi:10.1371/journal.pone.0241960)

**a**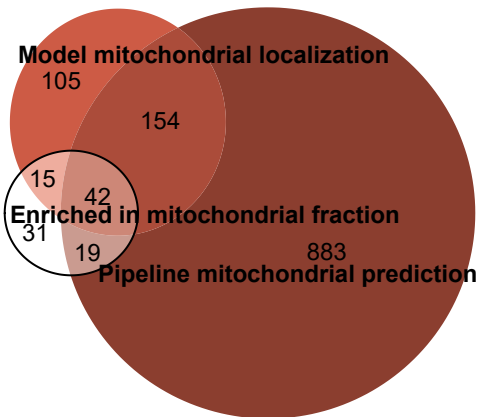**b**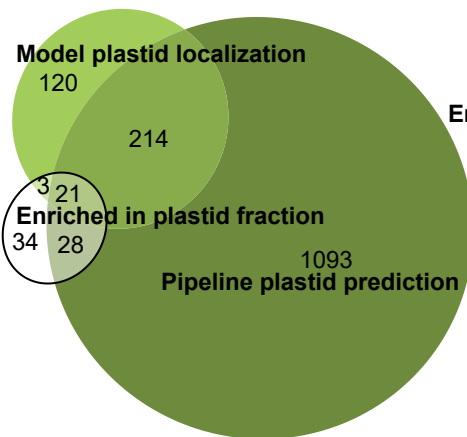**c**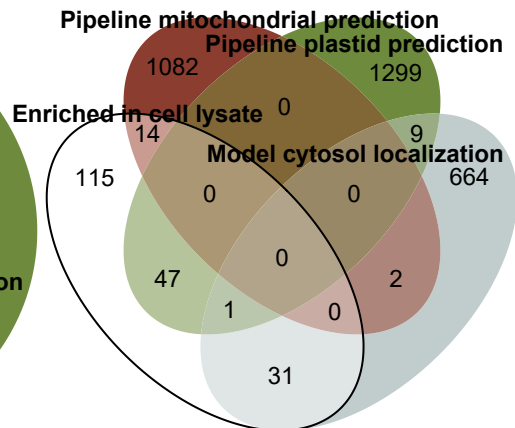

Supplement: S1 Fig — (PDF) [file pone.0241960.s001.pdf]

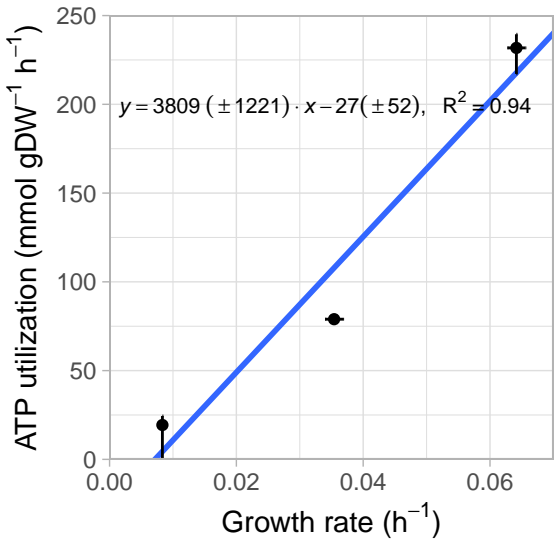

Supplement: S3 Fig — The x error bars represent the 95% confidence interval of measured dilution rates, and the y error bars follow from the 95% confidence intervals of bulk biomass measurements (carbohydrates, protein, total dry weight, in pg C/cell). (PDF) [file pone.0241960.s003.pdf]

Biomass composition (% deviation from target)

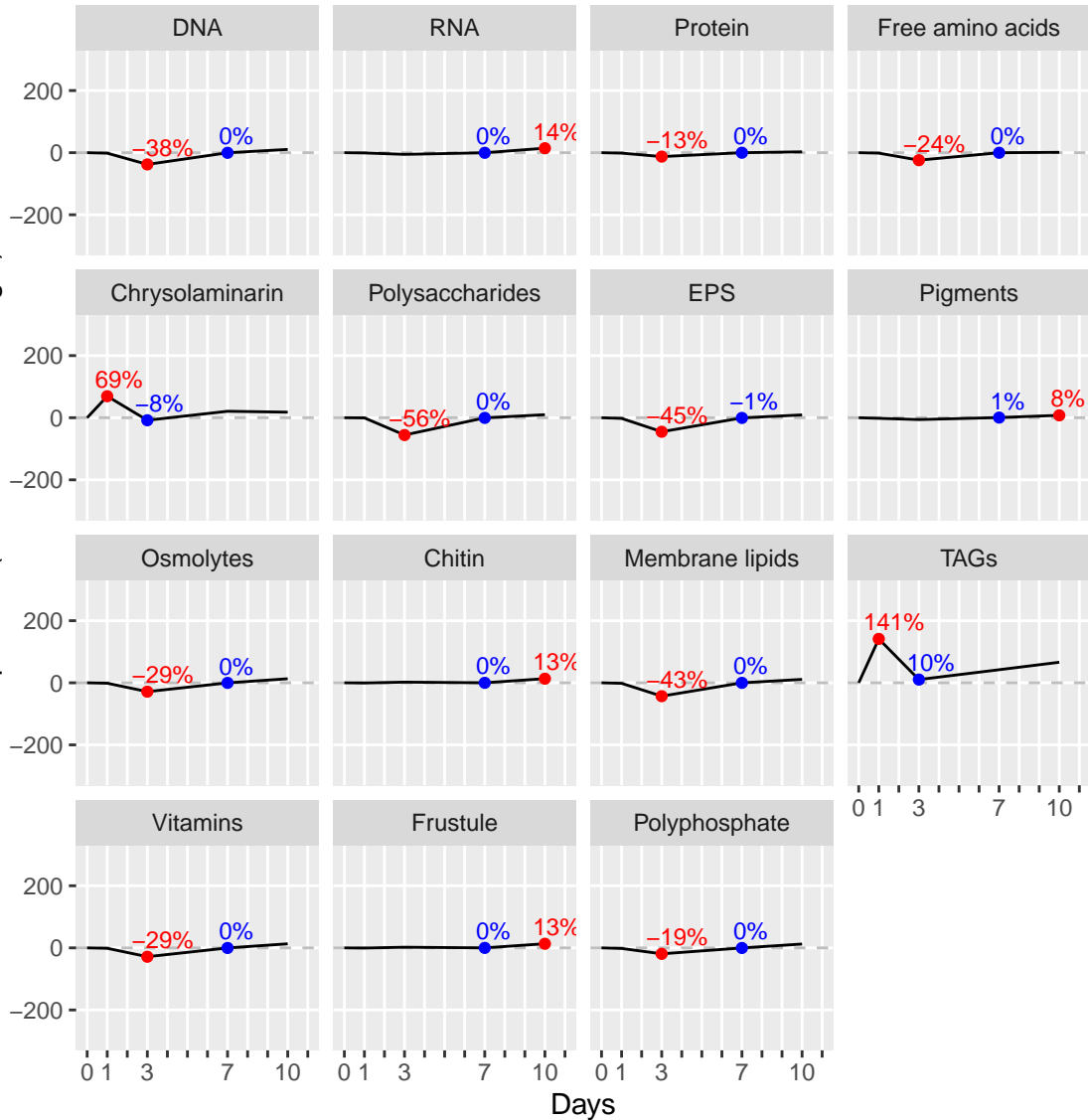

Supplement: S4 Fig — The best and worst (blue, red) estimates of biomass composition were marked for each biomass component. (A) Bulk biomass components, (B) RNA nucleotides, (C) Protein amino acids, (D) Free amino acids, (E) EPS sugars, (F) Pigment molecules, (G) sulfolipids, (H) phosphtidylcholines, (I) phosphatidylethanolamines, (J) phosphatidylglycerols, (K) diacylglycerides, (L) monogalactosyldiacylglycerides, (M) digalactosyldiacylglycerides, (N) triacylglycerides, (O) mg chl a /gDW, and (P) comparison of simulated elemental composition versus measured composition over the course of the experiment (days 0, 1, 2, 3, 5, 7, 10). (ZIP) [file pone.0241960.s004.zip › S4_Figs/S4A_Fig.pdf]

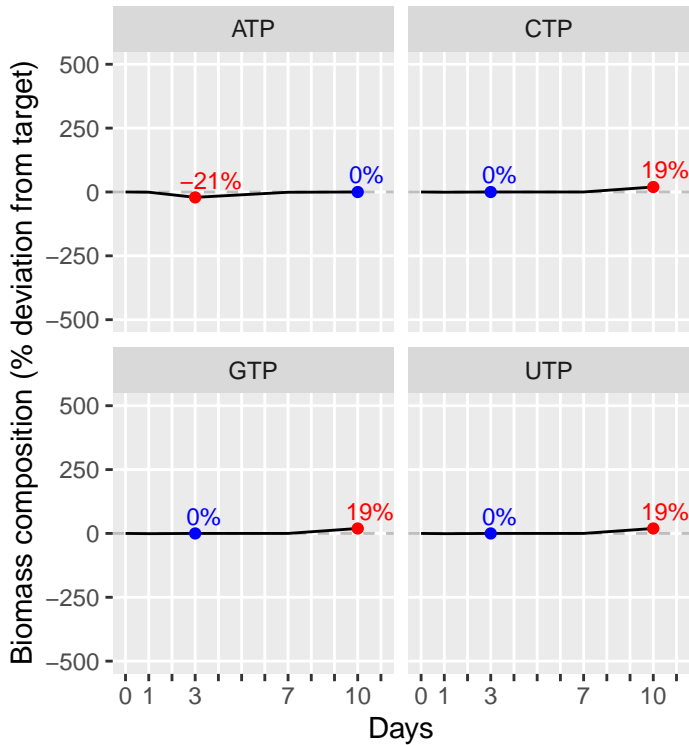

Supplement: S4 Fig — The best and worst (blue, red) estimates of biomass composition were marked for each biomass component. (A) Bulk biomass components, (B) RNA nucleotides, (C) Protein amino acids, (D) Free amino acids, (E) EPS sugars, (F) Pigment molecules, (G) sulfolipids, (H) phosphtidylcholines, (I) phosphatidylethanolamines, (J) phosphatidylglycerols, (K) diacylglycerides, (L) monogalactosyldiacylglycerides, (M) digalactosyldiacylglycerides, (N) triacylglycerides, (O) mg chl a /gDW, and (P) comparison of simulated elemental composition versus measured composition over the course of the experiment (days 0, 1, 2, 3, 5, 7, 10). (ZIP) [file pone.0241960.s004.zip › S4_Figs/S4B_Fig.pdf]

Biomass composition (% deviation from target)

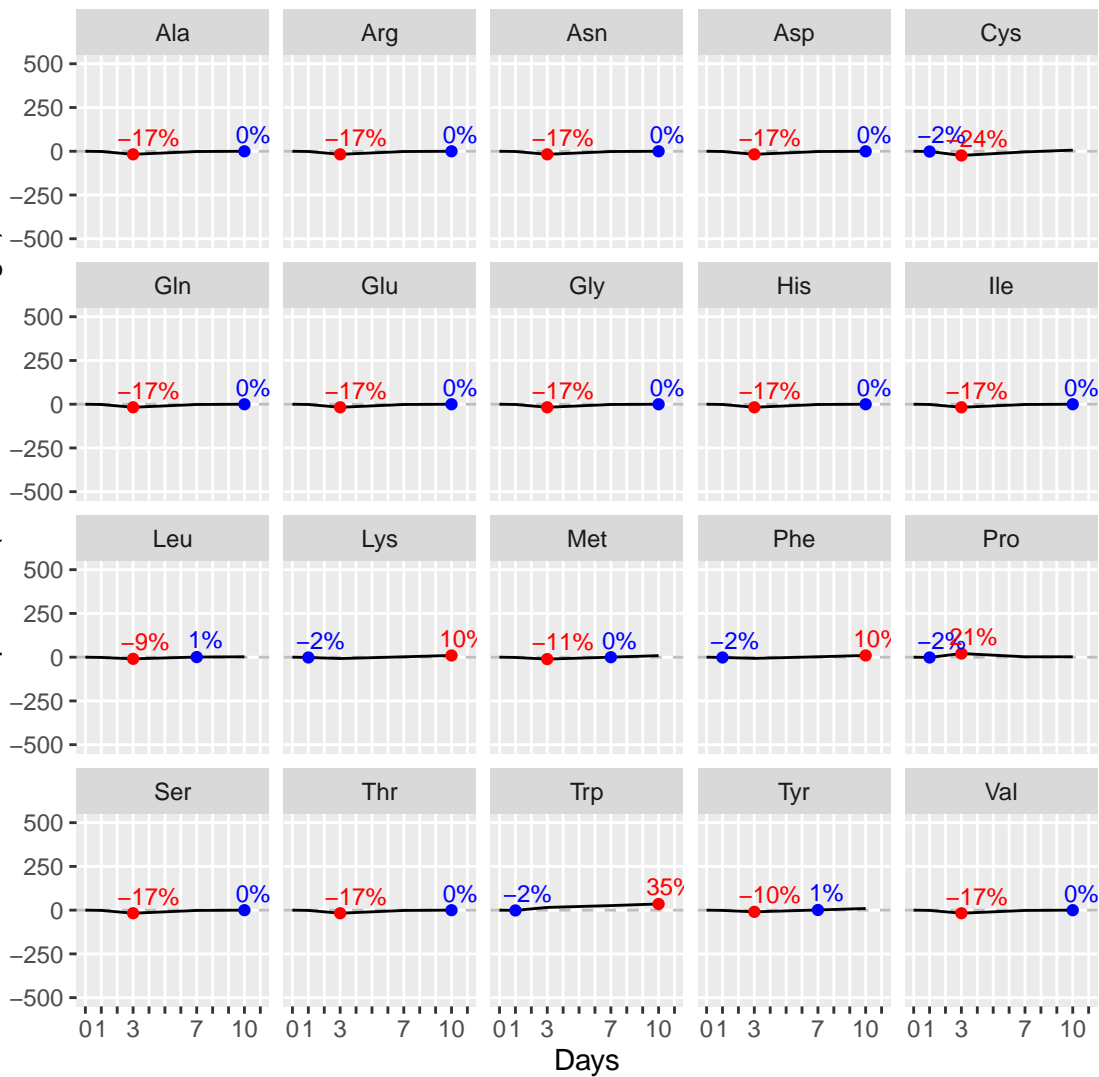

Supplement: S4 Fig — The best and worst (blue, red) estimates of biomass composition were marked for each biomass component. (A) Bulk biomass components, (B) RNA nucleotides, (C) Protein amino acids, (D) Free amino acids, (E) EPS sugars, (F) Pigment molecules, (G) sulfolipids, (H) phosphtidylcholines, (I) phosphatidylethanolamines, (J) phosphatidylglycerols, (K) diacylglycerides, (L) monogalactosyldiacylglycerides, (M) digalactosyldiacylglycerides, (N) triacylglycerides, (O) mg chl a /gDW, and (P) comparison of simulated elemental composition versus measured composition over the course of the experiment (days 0, 1, 2, 3, 5, 7, 10). (ZIP) [file pone.0241960.s004.zip › S4_Figs/S4C_Fig.pdf]

Biomass composition (% deviation from target)

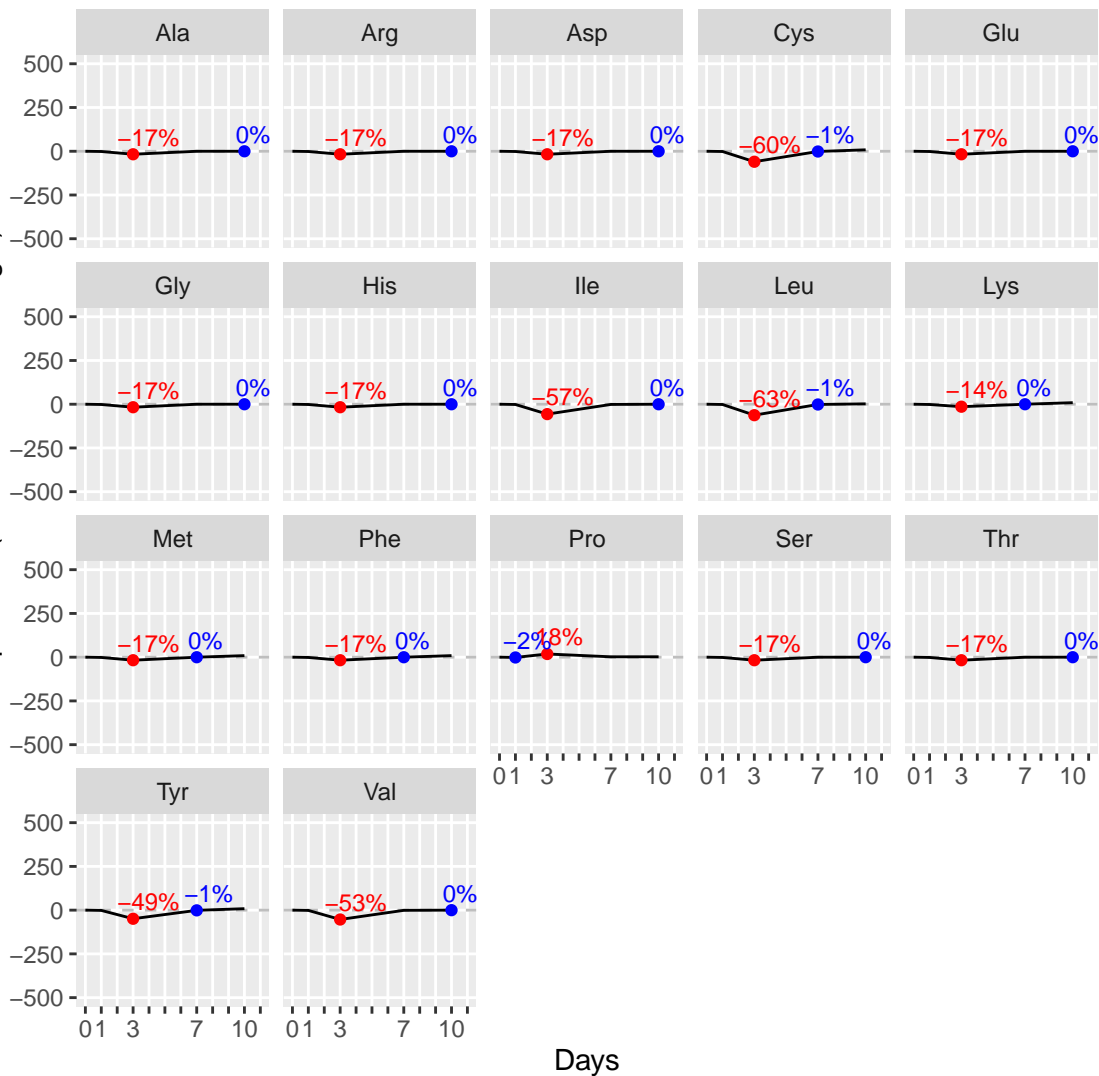

Supplement: S4 Fig — The best and worst (blue, red) estimates of biomass composition were marked for each biomass component. (A) Bulk biomass components, (B) RNA nucleotides, (C) Protein amino acids, (D) Free amino acids, (E) EPS sugars, (F) Pigment molecules, (G) sulfolipids, (H) phosphtidylcholines, (I) phosphatidylethanolamines, (J) phosphatidylglycerols, (K) diacylglycerides, (L) monogalactosyldiacylglycerides, (M) digalactosyldiacylglycerides, (N) triacylglycerides, (O) mg chl a /gDW, and (P) comparison of simulated elemental composition versus measured composition over the course of the experiment (days 0, 1, 2, 3, 5, 7, 10). (ZIP) [file pone.0241960.s004.zip › S4_Figs/S4D_Fig.pdf]

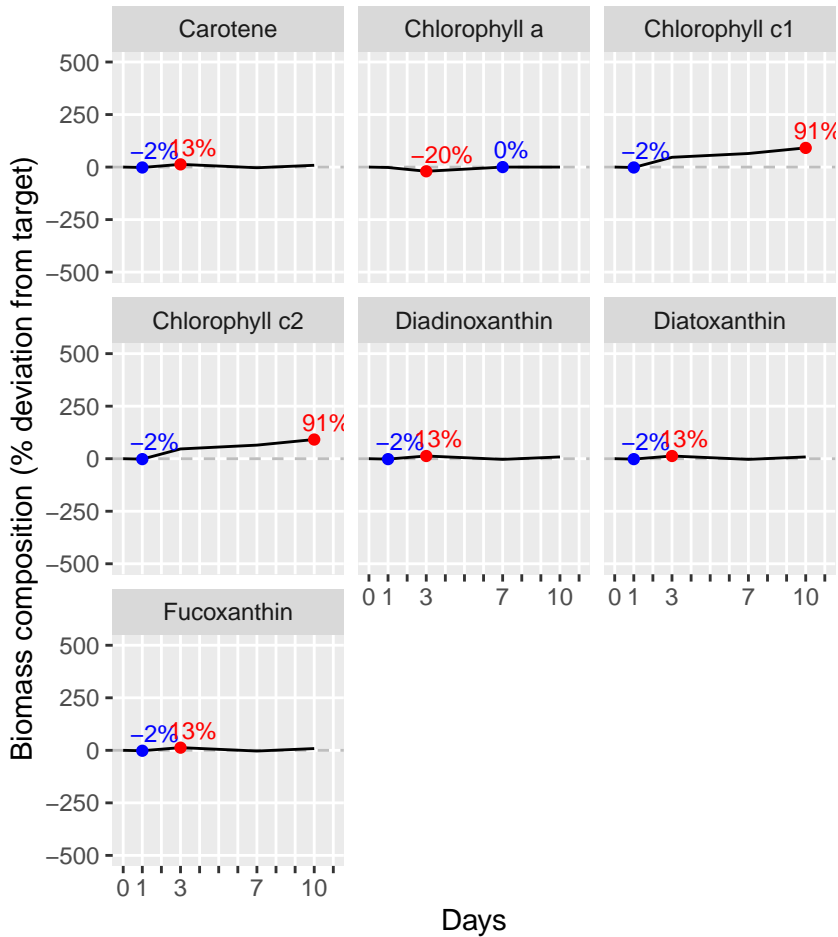

Supplement: S4 Fig — The best and worst (blue, red) estimates of biomass composition were marked for each biomass component. (A) Bulk biomass components, (B) RNA nucleotides, (C) Protein amino acids, (D) Free amino acids, (E) EPS sugars, (F) Pigment molecules, (G) sulfolipids, (H) phosphtidylcholines, (I) phosphatidylethanolamines, (J) phosphatidylglycerols, (K) diacylglycerides, (L) monogalactosyldiacylglycerides, (M) digalactosyldiacylglycerides, (N) triacylglycerides, (O) mg chl a /gDW, and (P) comparison of simulated elemental composition versus measured composition over the course of the experiment (days 0, 1, 2, 3, 5, 7, 10). (ZIP) [file pone.0241960.s004.zip › S4_Figs/S4F_Fig.pdf]

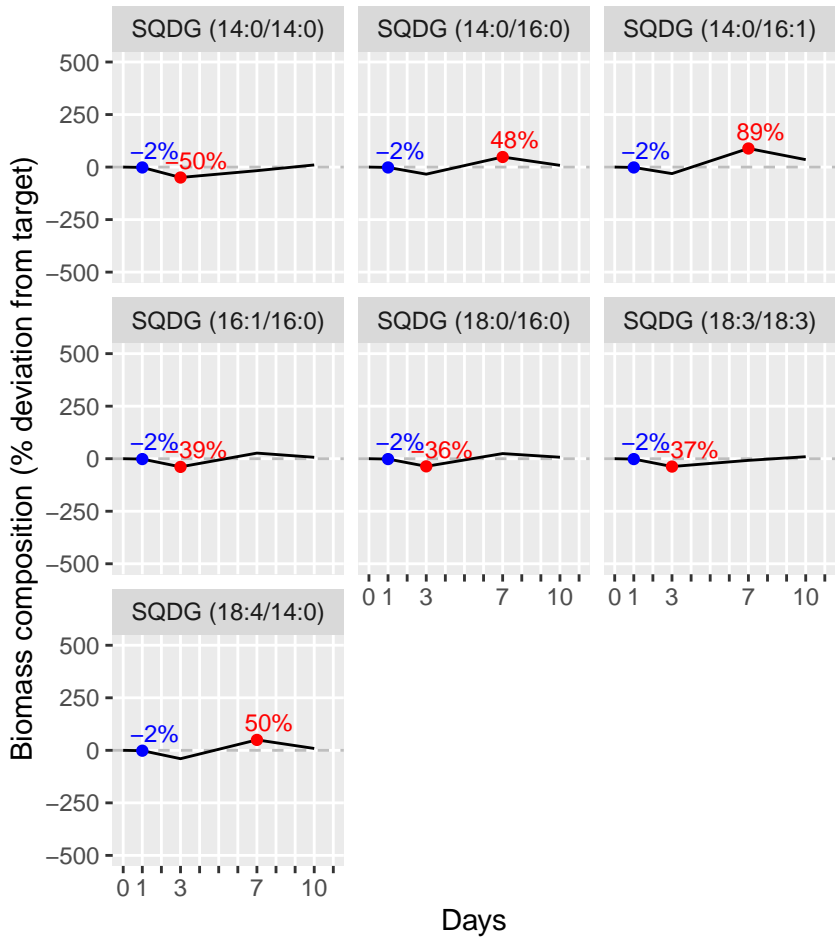

Supplement: S4 Fig — The best and worst (blue, red) estimates of biomass composition were marked for each biomass component. (A) Bulk biomass components, (B) RNA nucleotides, (C) Protein amino acids, (D) Free amino acids, (E) EPS sugars, (F) Pigment molecules, (G) sulfolipids, (H) phosphtidylcholines, (I) phosphatidylethanolamines, (J) phosphatidylglycerols, (K) diacylglycerides, (L) monogalactosyldiacylglycerides, (M) digalactosyldiacylglycerides, (N) triacylglycerides, (O) mg chl a /gDW, and (P) comparison of simulated elemental composition versus measured composition over the course of the experiment (days 0, 1, 2, 3, 5, 7, 10). (ZIP) [file pone.0241960.s004.zip › S4_Figs/S4G_Fig.pdf]

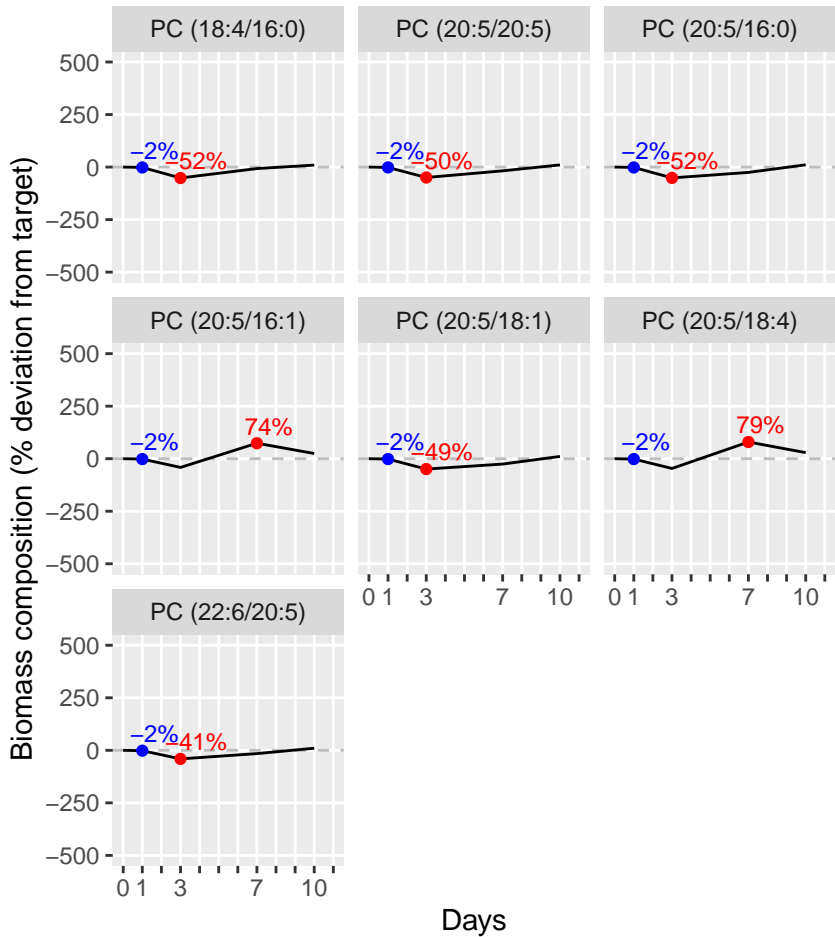

Supplement: S4 Fig — The best and worst (blue, red) estimates of biomass composition were marked for each biomass component. (A) Bulk biomass components, (B) RNA nucleotides, (C) Protein amino acids, (D) Free amino acids, (E) EPS sugars, (F) Pigment molecules, (G) sulfolipids, (H) phosphtidylcholines, (I) phosphatidylethanolamines, (J) phosphatidylglycerols, (K) diacylglycerides, (L) monogalactosyldiacylglycerides, (M) digalactosyldiacylglycerides, (N) triacylglycerides, (O) mg chl a /gDW, and (P) comparison of simulated elemental composition versus measured composition over the course of the experiment (days 0, 1, 2, 3, 5, 7, 10). (ZIP) [file pone.0241960.s004.zip › S4_Figs/S4H_Fig.pdf]

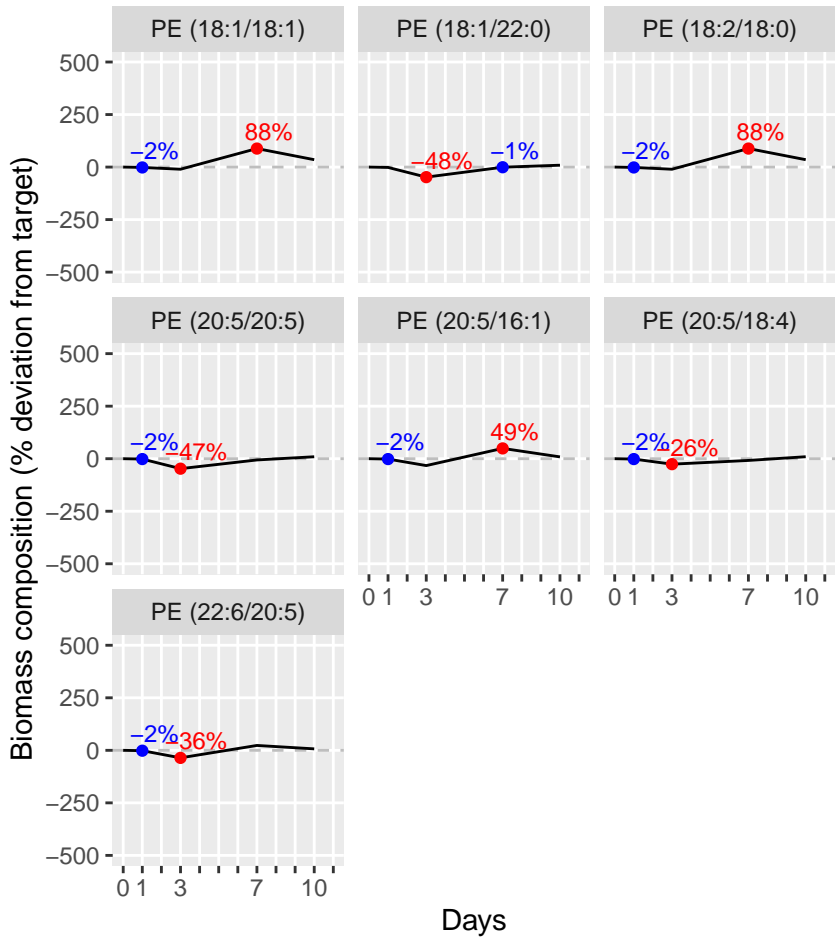

Supplement: S4 Fig — The best and worst (blue, red) estimates of biomass composition were marked for each biomass component. (A) Bulk biomass components, (B) RNA nucleotides, (C) Protein amino acids, (D) Free amino acids, (E) EPS sugars, (F) Pigment molecules, (G) sulfolipids, (H) phosphtidylcholines, (I) phosphatidylethanolamines, (J) phosphatidylglycerols, (K) diacylglycerides, (L) monogalactosyldiacylglycerides, (M) digalactosyldiacylglycerides, (N) triacylglycerides, (O) mg chl a /gDW, and (P) comparison of simulated elemental composition versus measured composition over the course of the experiment (days 0, 1, 2, 3, 5, 7, 10). (ZIP) [file pone.0241960.s004.zip › S4_Figs/S4I_Fig.pdf]

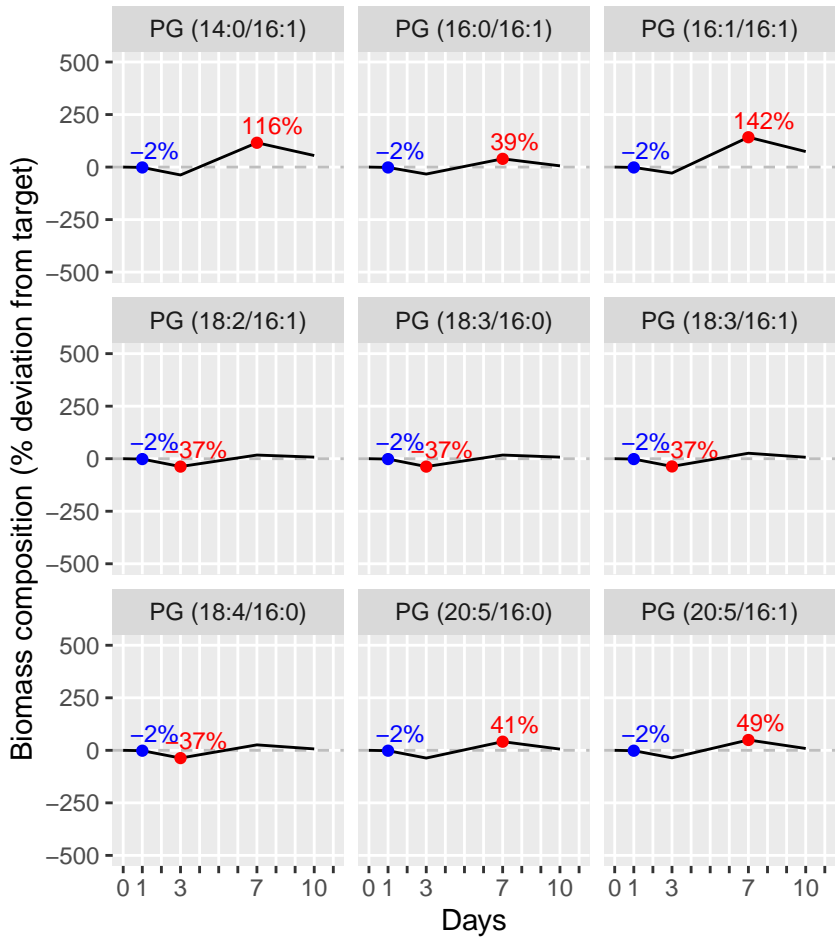

Supplement: S4 Fig — The best and worst (blue, red) estimates of biomass composition were marked for each biomass component. (A) Bulk biomass components, (B) RNA nucleotides, (C) Protein amino acids, (D) Free amino acids, (E) EPS sugars, (F) Pigment molecules, (G) sulfolipids, (H) phosphtidylcholines, (I) phosphatidylethanolamines, (J) phosphatidylglycerols, (K) diacylglycerides, (L) monogalactosyldiacylglycerides, (M) digalactosyldiacylglycerides, (N) triacylglycerides, (O) mg chl a /gDW, and (P) comparison of simulated elemental composition versus measured composition over the course of the experiment (days 0, 1, 2, 3, 5, 7, 10). (ZIP) [file pone.0241960.s004.zip › S4_Figs/S4J_Fig.pdf]

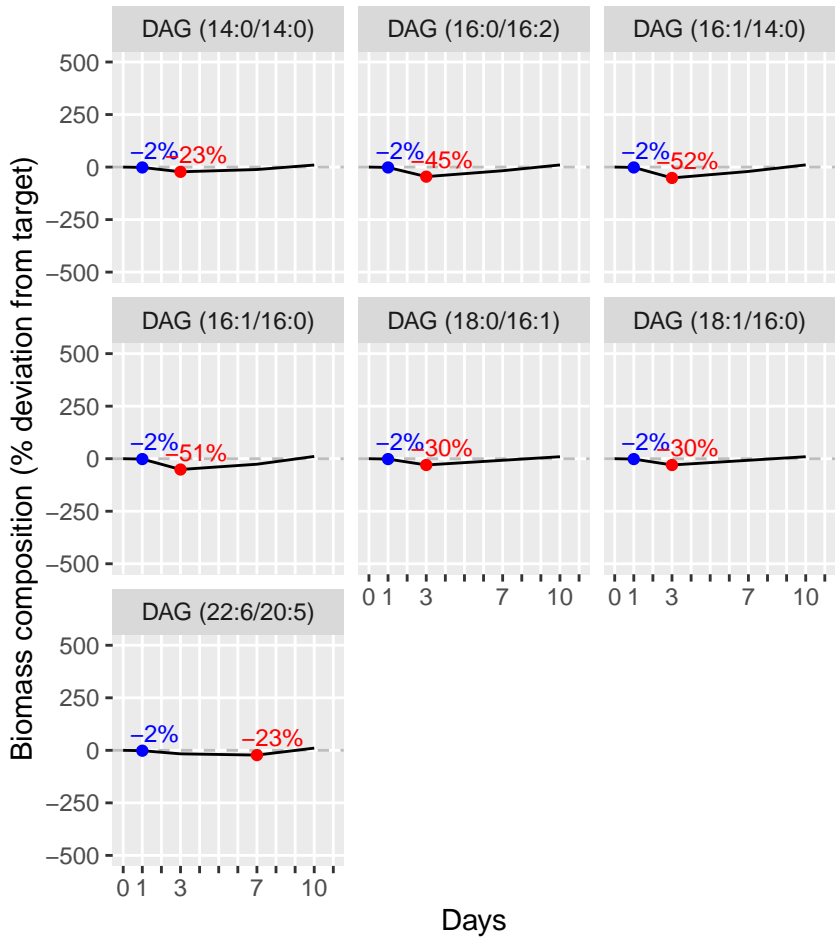

Supplement: S4 Fig — The best and worst (blue, red) estimates of biomass composition were marked for each biomass component. (A) Bulk biomass components, (B) RNA nucleotides, (C) Protein amino acids, (D) Free amino acids, (E) EPS sugars, (F) Pigment molecules, (G) sulfolipids, (H) phosphtidylcholines, (I) phosphatidylethanolamines, (J) phosphatidylglycerols, (K) diacylglycerides, (L) monogalactosyldiacylglycerides, (M) digalactosyldiacylglycerides, (N) triacylglycerides, (O) mg chl a /gDW, and (P) comparison of simulated elemental composition versus measured composition over the course of the experiment (days 0, 1, 2, 3, 5, 7, 10). (ZIP) [file pone.0241960.s004.zip › S4_Figs/S4K_Fig.pdf]

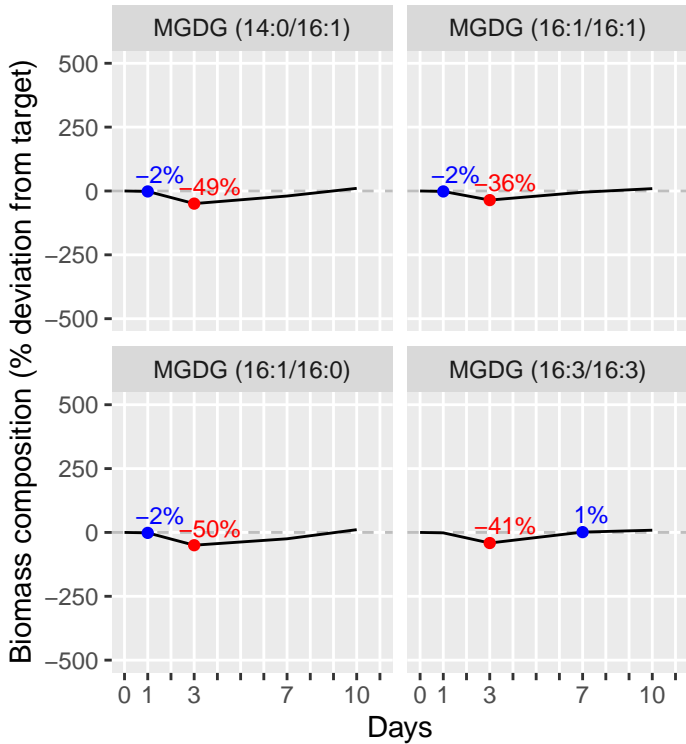

Supplement: S4 Fig — The best and worst (blue, red) estimates of biomass composition were marked for each biomass component. (A) Bulk biomass components, (B) RNA nucleotides, (C) Protein amino acids, (D) Free amino acids, (E) EPS sugars, (F) Pigment molecules, (G) sulfolipids, (H) phosphtidylcholines, (I) phosphatidylethanolamines, (J) phosphatidylglycerols, (K) diacylglycerides, (L) monogalactosyldiacylglycerides, (M) digalactosyldiacylglycerides, (N) triacylglycerides, (O) mg chl a /gDW, and (P) comparison of simulated elemental composition versus measured composition over the course of the experiment (days 0, 1, 2, 3, 5, 7, 10). (ZIP) [file pone.0241960.s004.zip › S4_Figs/S4L_Fig.pdf]

Biomass composition (% deviation from target)

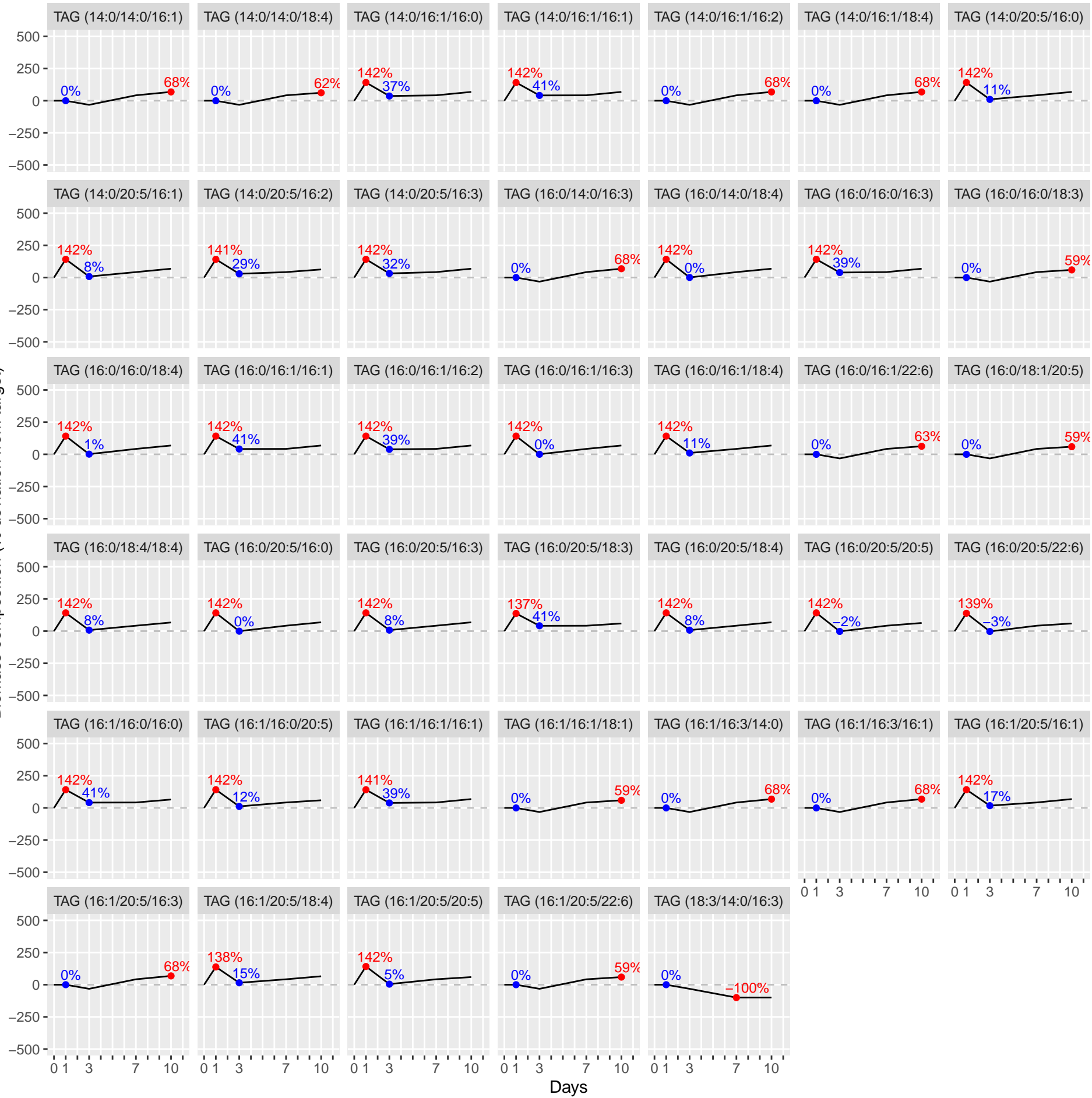

Supplement: S4 Fig — The best and worst (blue, red) estimates of biomass composition were marked for each biomass component. (A) Bulk biomass components, (B) RNA nucleotides, (C) Protein amino acids, (D) Free amino acids, (E) EPS sugars, (F) Pigment molecules, (G) sulfolipids, (H) phosphtidylcholines, (I) phosphatidylethanolamines, (J) phosphatidylglycerols, (K) diacylglycerides, (L) monogalactosyldiacylglycerides, (M) digalactosyldiacylglycerides, (N) triacylglycerides, (O) mg chl a /gDW, and (P) comparison of simulated elemental composition versus measured composition over the course of the experiment (days 0, 1, 2, 3, 5, 7, 10). (ZIP) [file pone.0241960.s004.zip › S4_Figs/S4N_Fig.pdf]

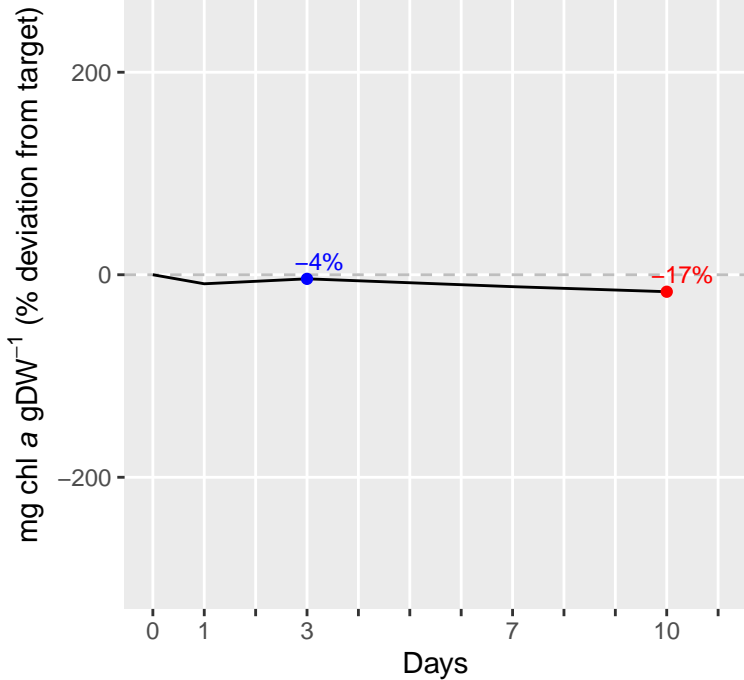

Supplement: S4 Fig — The best and worst (blue, red) estimates of biomass composition were marked for each biomass component. (A) Bulk biomass components, (B) RNA nucleotides, (C) Protein amino acids, (D) Free amino acids, (E) EPS sugars, (F) Pigment molecules, (G) sulfolipids, (H) phosphtidylcholines, (I) phosphatidylethanolamines, (J) phosphatidylglycerols, (K) diacylglycerides, (L) monogalactosyldiacylglycerides, (M) digalactosyldiacylglycerides, (N) triacylglycerides, (O) mg chl a /gDW, and (P) comparison of simulated elemental composition versus measured composition over the course of the experiment (days 0, 1, 2, 3, 5, 7, 10). (ZIP) [file pone.0241960.s004.zip › S4_Figs/S4O_Fig.pdf]

Elemental composition (% deviation from target)

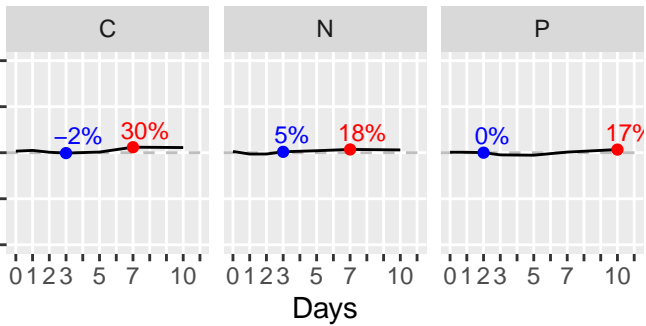

Supplement: S4 Fig — The best and worst (blue, red) estimates of biomass composition were marked for each biomass component. (A) Bulk biomass components, (B) RNA nucleotides, (C) Protein amino acids, (D) Free amino acids, (E) EPS sugars, (F) Pigment molecules, (G) sulfolipids, (H) phosphtidylcholines, (I) phosphatidylethanolamines, (J) phosphatidylglycerols, (K) diacylglycerides, (L) monogalactosyldiacylglycerides, (M) digalactosyldiacylglycerides, (N) triacylglycerides, (O) mg chl a /gDW, and (P) comparison of simulated elemental composition versus measured composition over the course of the experiment (days 0, 1, 2, 3, 5, 7, 10). (ZIP) [file pone.0241960.s004.zip › S4_Figs/S4P_Fig.pdf]
