# Supplementary material for: Genome-scale metabolic model of the diatom Thalassiosira pseudonana highlights the importance of nitrogen and sulfur metabolism in redox balance
Source: PLoS One. 2021 Mar 24;16(3):e0241960. doi: 10.1371/journal.pone.0241960 (PMC7990286; doi:10.1371/journal.pone.0241960)
Supplement: S4 Fig — The best and worst (blue, red) estimates of biomass composition were marked for each biomass component. (A) Bulk biomass components, (B) RNA nucleotides, (C) Protein amino acids, (D) Free amino acids, (E) EPS sugars, (F) Pigment molecules, (G) sulfolipids, (H) phosphtidylcholines, (I) phosphatidylethanolamines, (J) phosphatidylglycerols, (K) diacylglycerides, (L) monogalactosyldiacylglycerides, (M) digalactosyldiacylglycerides, (N) triacylglycerides, (O) mg chl a /gDW, and (P) comparison of simulated elemental composition versus measured composition over the course of the experiment (days 0, 1, 2, 3, 5, 7, 10). (ZIP) [file pone.0241960.s004.zip › S4_Figs/S4E_Fig.pdf]

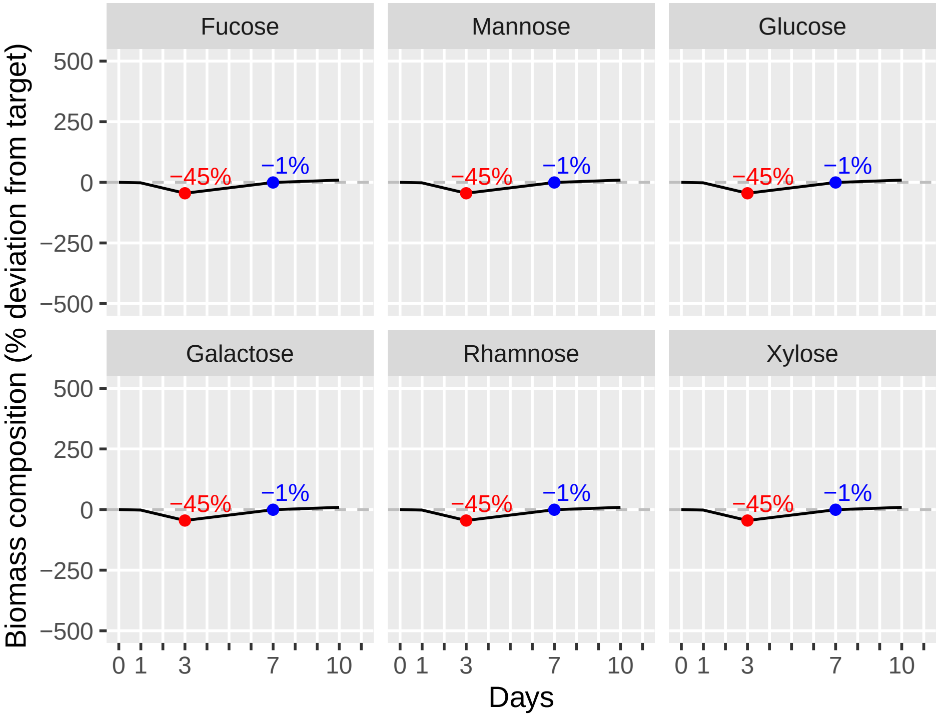
Fucose
Mannose
Glucose
Galactose
Rhamnose
Xylose
−45%
−1%
500
250
0
−250
−500
0 1 3 7 10
Days
Biomass composition (% deviation from target)
