# Supplementary material for: Genome-scale metabolic model of the diatom Thalassiosira pseudonana highlights the importance of nitrogen and sulfur metabolism in redox balance
Source: PLoS One. 2021 Mar 24;16(3):e0241960. doi: 10.1371/journal.pone.0241960 (PMC7990286; doi:10.1371/journal.pone.0241960)
Supplement: S1 Table — (DOCX) [file pone.0241960.s005.docx]

| **Table S1** Comparison of attributes of *i*Tps1432 and *P. tricornutum* GEMs | | | |
| --- | --- | --- | --- |
| **Property** | ***Thalasssiosira pseudonana*** **CCMP 1335** | ***Phaeodactylum tricornutum* CCAP 1055/1** | |
| **Model name** | *i*Tps1432 | *i*LB1034 (Broddrick *et al.*, 2019) | *i*LB1027_lipid (Levering *et al.*, 2016) |
| **Genes**  **Total**  Included in models  Complexed^a^ | 11,849 (Armbrust *et al*., 2004)  13,344 (Gruber *et al.*, 2015)  1,432 (12.09% / 10.73%)  182 | 10,402 (Bowler *et al.*, 2008)  1,034 (9.94%)  172 | 10,402 (Bowler *et al.*, 2008)  1,032 (9.92%)  172 |
| **Reactions**  Reversible  Irreversible  Gene associated  Non-gene associated  Metabolic  Transport  Demand^b^  Sink^c^  Exchange^d^  Biomass  Extracellular  Cytoplasm  Plastid  Thylakoid lumen  Mitochondria  Peroxisome  **Total**  Unique  Blocked^e^  Orphaned^f^ | 633  5,446  5,578  501  5,553  432  3  1  72  16  126  4,365  900  12  531  145  6,079  5,627  545  0 | 423  1,739  1,861  301  1,801  297  16  1  30  13  51  994  441  7  530  138  2,162  1,869  487  4 | 423  4,033  4,150  306  4,093  308  13  1  30  8  51  3,078  657  7  525  138  4,456  4,130  381  4 |
| **Metabolites**  Extracellular  Cytoplasm  Plastid  Thylakoid lumen  Mitochondria  Peroxisome  **Total**  Unique  Dead-ends^g^ | 73  1,520  566  15  477  141  2,792  2,007  425 | 30  713  384  9  447  131  1,714  1,153  446 | 30  1,130  428  9  443  132  2,172  1,583  340 |

^a^ Complexed genes are those that together encode multiple subunits of an enzyme. They are denoted as complexes in the gene reaction rules using the word ‘and’, while genes performing the same reaction are connected by the word ‘or’.

^b^ Demand reactions are unbalanced reactions (have substrates but no products). These reactions deal with metabolites that are known to be produced (and not consumed) but have no degradation pathway, are not substrates in the biomass reaction, and are not known to be transported out of the cell, for example loss of photons (as heat or fluorescence).

c Sink reactions are similar to demand reactions but are reversible. These reactions are a source and sink for metabolites that are required by the model but are not part of the extracellular environment, for example a protein-linked asparagine residue required for N-glycosylation.

^d^ Exchange reactions are unbalanced extracellular reactions that are used to control the supply or removal of metabolites in the media.

^e^ Blocked reactions are reactions that cannot carry flux due to missing reactions in the network.

^f^ Orphaned reactions are blocked reactions that are disconnected from the entire network.

^g^ Dead-end metabolites are metabolites that are only linked to blocked reactions.
