# Supplementary material for: Genome-scale metabolic model of the diatom Thalassiosira pseudonana highlights the importance of nitrogen and sulfur metabolism in redox balance
Source: PLoS One. 2021 Mar 24;16(3):e0241960. doi: 10.1371/journal.pone.0241960 (PMC7990286; doi:10.1371/journal.pone.0241960)
Supplement: S2 Table — (DOCX) [file pone.0241960.s006.docx]

| **Table S2** Transporters added to *i*Tps1432 and associated evidence^1^ | | | | |
| --- | --- | --- | --- | --- |
| **Reaction ID** | **Reaction name** | **Gene-reaction rule** | **Evidence** | **Evidence type** |
| GLUt2r_e | L-Glutamate via proton symport |  | Glu/Asp transporters in R. pomeroyi upregulated in co-culture with T. pseudonana (10.1111/1462-2920.13834) | Biochemical |
| ASPt2r_e | L-Aspartate via proton symport |  | Glu/Asp transporters in R. pomeroyi upregulated in co-culture with T. pseudonana (10.1111/1462-2920.13834) | Biochemical |
| CNCBL3t_e | Cyanocob(III)alamin transporter | Thaps3a_11697 | Gene identified as likely cobalamin transporter (10.1073/pnas.1201731109) | Biochemical |
| AQCOBALt_e | Aquacob(III)alamin transporter | Thaps3a_11697 | Gene identified as likely cobalamin transporter (10.1073/pnas.1201731109) | Biochemical |
| NACTAURt_e | N-acetyltaurine via proton symport | Thaps3a_23412 | N-acetyltaurine transporter in R. pomeroyi upregulated in co-culture with T. pseudonana (10.1111/1462-2920.13834) | Biochemical |
| UACGAMt_e | UDP-N-acetyl-alpha-D-glucosamine transport, extracellular | Thaps3a_2088 or Thaps3u_393 | UDP-N-acetyl-glucosamine transporter in R. pomeroyi upregulated in co-culture with T. pseudonana (10.1111/1462-2920.13834) | Biochemical |
| DHPSt_e | Dihydroxypropanesulfonate transport via diffusion | Thaps3a_23412 | DHPS transporter in R. pomeroyi upregulated in co-culture with T. pseudonana (10.1111/1462-2920.13834) | Biochemical |
| SIO4H4t_e | Silicic acid transport, extracellular | THAPSDRAFT_268895 or THAPS_41392 or THAPSDRAFT_35133 | Genes identified as a silicon transporters (10.1111/jpy.12441) | Biochemical |
| BTNt2_e | Biotin transport |  | Many organisms transport biotin | Physiology |
| DMSPt_e | Dimethylsulfoniopropanoate via proton symport | Thaps3a_22440 | T. pseudonana can import DMSP and there is evidence that glycine betaine transporters can also transport DMSP (10.1126/science.1131043) | Physiology |
| GLYBt_e | Glycine betaine via proton symport | Thaps3a_22440 or Thaps3a_3793 | T. pseudonana can import glycine betaine (10.1126/science.1131043) | Physiology |
| ATPt_e | ATP transport, extracellular |  | T. pseudonana can grow on ATP as a sole source of phosphorus (10.1111/1462-2920.14630) | Physiology |
| AMPt_e | AMP transport, extracellular |  | T. pseudonana can grow on AMP as a sole source of phosphorus (10.1111/1462-2920.14630) | Physiology |
| PPPIt_e | Triphosphate transport, extracellular |  | T. pseudonana can grow on triphosphate as a sole source of phosphate (10.1111/1462-2920.14630) | Physiology |
| ILEt2r_e | L-Isoleucine via proton symport | Thaps3a_26635 | Certain free amino acids are excreted by a variety of diatoms (10.1016/0022-0981(86)90216-9), gene is annotated as AROMATIC AND NEUTRAL TRANSPORTER 1 | Sequence |
| LEUt2r_e | L-Leucine via proton symport | Thaps3a_26635 | Certain free amino acids are excreted by a variety of diatoms (10.1016/0022-0981(86)90216-9), gene is annotated as AROMATIC AND NEUTRAL TRANSPORTER 1 | Sequence |
| VALt2r_e | L-Valine via proton symport | Thaps3a_26635 | Certain free amino acids are excreted by a variety of diatoms (10.1016/0022-0981(86)90216-9), gene is annotated as AROMATIC AND NEUTRAL TRANSPORTER 1 | Sequence |
| ALANA1t_e | L-Alanine:Na+ symporter, extracellular | Thaps3u_463 or Thaps3a_265122 or Thaps3a_265120 or Thaps3a_263385 or Thaps3a_25394 or Thaps3a_23180 or Thaps3a_21347 or Thaps3a_20871 or Thaps3a_1200 | Certain free amino acids are excreted by a variety of diatoms (10.1016/0022-0981(86)90216-9), genes are annotated as SODIUM-COUPLED NEUTRAL AMINO ACID TRANSPORTER 11-RELATED and SODIUM-COUPLED NEUTRAL AMINO ACID TRANSPORTER 6-RELATED | Sequence |
| SERNA1t_e | L-Serine:Na+ symporter, extracellular | Thaps3u_463 or Thaps3a_265122 or Thaps3a_265120 or Thaps3a_263385 or Thaps3a_25394 or Thaps3a_23180 or Thaps3a_21347 or Thaps3a_20871 or Thaps3a_1200 | Certain free amino acids are excreted by a variety of diatoms (10.1016/0022-0981(86)90216-9), genes are annotated as SODIUM-COUPLED NEUTRAL AMINO ACID TRANSPORTER 11-RELATED and SODIUM-COUPLED NEUTRAL AMINO ACID TRANSPORTER 6-RELATED | Sequence |
| GLNNA1t_e | L-Glutamine:Na+ symporter, extracellular | Thaps3u_463 or Thaps3a_265122 or Thaps3a_265120 or Thaps3a_263385 or Thaps3a_25394 or Thaps3a_23180 or Thaps3a_21347 or Thaps3a_20871 or Thaps3a_1200 | Certain free amino acids are excreted by a variety of diatoms (10.1016/0022-0981(86)90216-9), genes are annotated as SODIUM-COUPLED NEUTRAL AMINO ACID TRANSPORTER 11-RELATED and SODIUM-COUPLED NEUTRAL AMINO ACID TRANSPORTER 6-RELATED | Sequence |
| HISNA1t_e | L-Histidine:Na+ symporter, extracellular | Thaps3u_463 or Thaps3a_265122 or Thaps3a_265120 or Thaps3a_263385 or Thaps3a_25394 or Thaps3a_23180 or Thaps3a_21347 or Thaps3a_20871 or Thaps3a_1200 | Certain free amino acids are excreted by a variety of diatoms (10.1016/0022-0981(86)90216-9), genes are annotated as SODIUM-COUPLED NEUTRAL AMINO ACID TRANSPORTER 11-RELATED and SODIUM-COUPLED NEUTRAL AMINO ACID TRANSPORTER 6-RELATED | Sequence |
| ASNNA1t_e | L-Asparagine:Na+ symporter, extracellular | Thaps3u_463 or Thaps3a_265122 or Thaps3a_265120 or Thaps3a_263385 or Thaps3a_25394 or Thaps3a_23180 or Thaps3a_21347 or Thaps3a_20871 or Thaps3a_1200 | Certain free amino acids are excreted by a variety of diatoms (10.1016/0022-0981(86)90216-9), genes are annotated as SODIUM-COUPLED NEUTRAL AMINO ACID TRANSPORTER 11-RELATED and SODIUM-COUPLED NEUTRAL AMINO ACID TRANSPORTER 6-RELATED | Sequence |
| THRt2r_e | L-Threonine via proton symport |  | Certain free amino acids are excreted by a variety of diatoms (10.1016/0022-0981(86)90216-9) | Sequence |
| GLYt2r_e | Glycine via proton symport |  | Certain free amino acids are excreted by a variety of diatoms (10.1016/0022-0981(86)90216-9) | Sequence |
| PROLt_e | L-Proline via proton symport | Thaps3a_22440 or Thaps3a_3793 | Genes annotated as PROLINE/BETAINE TRANSPORTER | Sequence |
| URAt_e | Uracil permease, extracellular | Thaps3a_30765 or Thaps3a_12056_25851 or Thaps3a_267876 or Thaps3a_260657 | Genes annotated as xanthine/uracil permease | Sequence |
| XANt_e | Xanthine permease, extracellular | Thaps3a_30765 or Thaps3a_12056_25851 or Thaps3a_267876 or Thaps3a_260657 | Genes annotated as xanthine/uracil permease | Sequence |
| CHOLNAt_e | Choline, sodium cotransport | Thaps3a_5607 or Thaps3a_28183 or Thaps3a_5041_22552 or Thaps3a_20595 or Thaps3a_21480 or Thaps3a_22820 or Thaps3a_22830 or Thaps3a_22844 or Thaps3a_262743 or Thaps3a_22995 | Genes annotated as choline transporters, choline can be taken up by sea-ice diatoms (10.1111/jpy.12839) | Sequence |
| FRMDt_e | Formamide transport, extracellular |  | Reaction required to unblock histidine degradation to glutamate | Modeling |
| FORt_e | Formate transport, extracellular |  | Present in C. reinhardtii model, iRC1080 | Modeling |
| ACt_e | Acetate transporter, extracellular |  | Present in C. reinhardtii model, iRC1080 | Modeling |

^1^ Descriptions of evidence type are from Thiele & Palsson (2010), and are displayed in order of highest to lowest confidence.
