# Supplementary material for: Genome-scale metabolic model of the diatom Thalassiosira pseudonana highlights the importance of nitrogen and sulfur metabolism in redox balance
Source: PLoS One. 2021 Mar 24;16(3):e0241960. doi: 10.1371/journal.pone.0241960 (PMC7990286; doi:10.1371/journal.pone.0241960)
Supplement: S1 Note — (DOCX) [file pone.0241960.s011.docx]

**Supplementary Note S1**

Due to differences in reaction, metabolite, and gene IDs, we compared *i*Tps1432 and iThaps987 based on EC number. Ahmad, *et al.* (2020) also chose to compare *i*Thaps987 with *i*LB1025 based on EC number. We re-created the Venn diagram in their Figure 1A and found a slightly different result, where 488 EC numbers are shared (rather than 483), *i*Thaps987 has 200 unique EC numbers (rather than 183), and *i*LB1025 has 180 unique EC numbers (rather than 191). The source of this discrepancy is unclear, but these data were taken directly from the *i*Tps987 and *i*LB1025 Excel spreadsheets provided with each publication and the EC numbers were processed to remove any formatting errors present. When *i*Tps1432 was added to the comparison, we found that *i*Tps1432 had more overlap with *i*LB1025. The draft model of *i*Thaps987 was constructed from the BioCyc Pathway Genome Database (PGDB) for *Thalassiosira pseudonana*, while the draft model for *i*Tps1432 was constructed from the *i*LB1027_lipid model (a version of *i*LB1025 which includes lipid metabolism). The different re-construction methods and gene annotation sources are the likely cause of this result (see Griesemer, *et al*., 2018). Most genes present in *i*Thaps987 and not *i*LB1025 (and by extension *i*Tps1432) were discussed by Ahmad*, et al.* (2020). The 20 EC numbers present in *i*Tps1432 alone are listed below. Reconciling the two models could improve future iterations of *T. pseudonana* genome-scale metabolic models.

| **Reaction ID** | **Reaction name** | **EC number** |
| --- | --- | --- |
| DGDGH_MYRS_HDE_h | Galactolipid acylhydrolase (DGDG)(14:0/16:1(9Z)) | 3.1.1.28 |
| MGDGH_HDTE_HDTE_h | Galactolipid acylhydrolase (MGDG)(16:3(6Z,9Z,12Z)/16:3(6Z,9Z,12Z)) | 3.1.1.27 |
| DGDGH_PALM_HDDE_h | Galactolipid acylhydrolase (DGDG)(16:0/16:2(9Z,12Z)) | 3.1.1.29 |
| DGDGH_ALNA_PALM_h | Galactolipid acylhydrolase (DGDG)(18:3(9Z,12Z,15Z)/16:0) | 3.1.1.29 |
| DGDGH_GLNA_PALM_h | Galactolipid acylhydrolase (DGDG)(18:3(6Z,9Z,12Z)/16:0) | 3.1.1.29 |
| DGDGH_LNA_PALM_h | Galactolipid acylhydrolase (DGDG)(18:2(9Z,12Z)/16:0) | 3.1.1.29 |
| DGDGH_STA_PALM_h | Galactolipid acylhydrolase (DGDG)(18:4(6Z,9Z,12Z,15Z)/16:0) | 3.1.1.29 |
| DGDGH_ALNA_HDE_h | Galactolipid acylhydrolase (DGDG)(18:3(9Z,12Z,15Z)/16:1(9Z)) | 3.1.1.29 |
| DGDGH_GLNA_HDE_h | Galactolipid acylhydrolase (DGDG)(18:3(6Z,9Z,12Z)/16:1(9Z)) | 3.1.1.29 |
| DGDGH_LNA_HDE_h | Galactolipid acylhydrolase (DGDG)(18:2(9Z,12Z)/16:1(9Z)) | 3.1.1.29 |
| DGDGH_STA_HDE_h | Galactolipid acylhydrolase (DGDG)(18:4(6Z,9Z,12Z,15Z)/16:1(9Z)) | 3.1.1.29 |
| CNCBLR_c | Cyanocob(III)alamin reductase | 1.16.1.6 |
| CNCBL3t_e | Cyanocob(III)alamin transporter | 3.6.3.33 |
| AQCOBALt_e | Aquacob(III)alamin transporter | 3.6.3.33 |
| AQCOBALOR_c | NADPH:aquacob(III)alamin oxidoreductase | 1.16.1.5 |
| CBL2R_c | Cob(II)alamin reductase | 1.16.1.4 |
| CBLt_c | NADH:cob(II)alamin oxidoreductase | 1.16.1.4 |
| ACGAMK_c | N-acetylglucosamine kinase | 2.7.1.59 |
| KAS15_h | 3-oxoacyl-[acyl-carrier-protein] synthase III | 2.3.1.180 |
| GCPN_c | Guanosine 3',5'-cyclic phosphate 5'-nucleotidohydrolase | 3.1.4.17 |
| APN_c | Adenosine 3',5'-phosphate 5'-nucleotidohydrolase | 3.1.4.17 |
| MTRR_c | Methionine synthase reductase | 1.16.1.8 |
| SPD3DH_c | Sulfopropanediol 3-dehydrogenase | 1.1.1.308 |
| SERPTA_c | Serinol phosphate--pyruvate transaminase | 2.6.1.M7 |
| ADPRDP_h | ADP-ribose diphosphatase | 3.6.1.13 |
| ADPRDP_c | ADP-ribose diphosphatase | 3.6.1.13 |
| NADN_c | NAD nucleosidase | 3.2.2.5 |
| GLACASE_c | D-Glucono-1,5-lactone lactonohydrolase | 3.1.1.17 |
| G1D_c | Glucose 1-dehydrogenase | 1.1.1.118 |
| G1D_c | Glucose 1-dehydrogenase | 1.1.1.118 |
| CHITN2_c | Chitinase | 3.2.1.14 |
| HXAD_c | Hexosaminidase | 3.2.1.52 |


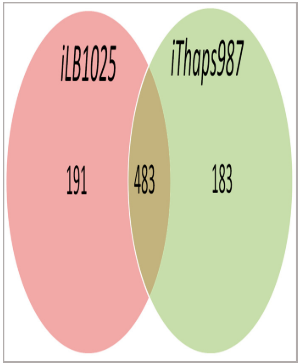


Figure 1A from Ahmad, *et al.* (2020)


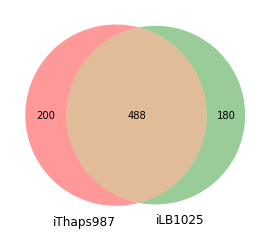

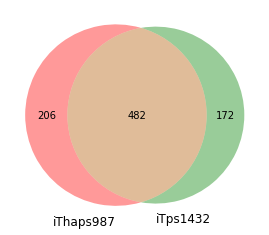

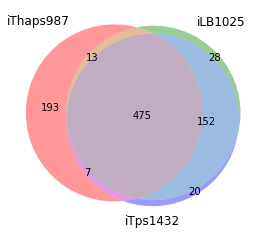


Re-production of Figure 1A and comparisons with *i*Tps1432.
