# Supplementary material for: Genome-scale metabolic model of the diatom Thalassiosira pseudonana highlights the importance of nitrogen and sulfur metabolism in redox balance
Source: PLoS One. 2021 Mar 24;16(3):e0241960. doi: 10.1371/journal.pone.0241960 (PMC7990286; doi:10.1371/journal.pone.0241960)
Supplement: S4 File — (DOCX) [file pone.0241960.s015.docx]

**Supplementary references**

Ahmad A, Tiwari A, Srivastava S. A Genome-Scale Metabolic Model of Thalassiosira pseudonana CCMP 1335 for a Systems-Level Understanding of Its Metabolism and Biotechnological Potential. Microorganisms. 2020;8: 1396. doi:10.3390/microorganisms8091396

Armbrust E V. The Genome of the Diatom Thalassiosira Pseudonana: Ecology, Evolution, and Metabolism. Science (80- ). 2004;306: 79–86. doi:10.1126/science.1101156

Bowler C, Allen AE, Badger JH, Grimwood J, Jabbari K, Kuo A, et al. The Phaeodactylum genome reveals the evolutionary history of diatom genomes. Nature. 2008;456: 239–244. doi:10.1038/nature07410

Broddrick JT, Du N, Smith SR, Tsuji Y, Jallet D, Ware MA, et al. Cross‐compartment metabolic coupling enables flexible photoprotective mechanisms in the diatom Phaeodactylum tricornutum. New Phytol. 2019;222: 1364–1379. doi:10.1111/nph.15685

Griesemer M, Kimbrel JA, Zhou CE, Navid A, D’Haeseleer P. Combining multiple functional annotation tools increases coverage of metabolic annotation. BMC Genomics. 2018;19: 1–11. doi:10.1186/s12864-018-5221-9

Gruber A, Rocap G, Kroth PG, Armbrust EV, Mock T. Plastid proteome prediction for diatoms and other algae with secondary plastids of the red lineage. Plant J. 2015;81: 519–528. doi:10.1111/tpj.12734

Levering J, Broddrick J, Dupont CL, Peers G, Beeri K, Mayers J, et al. Genome-Scale Model Reveals Metabolic Basis of Biomass Partitioning in a Model Diatom. Ianora A, editor. PLoS One. 2016;11: e0155038. doi:10.1371/journal.pone.0155038

Schober AF, Río Bártulos C, Bischoff A, Lepetit B, Gruber A, Kroth PG. Organelle Studies and Proteome Analyses of Mitochondria and Plastids Fractions from the Diatom Thalassiosira pseudonana. Plant Cell Physiol. 2019;60: 1811–1828. doi:10.1093/pcp/pcz097

Thiele I, Palsson BØ. A protocol for generating a high-quality genome-scale metabolic reconstruction. Nat Protoc. 2010;5: 93–121. doi:10.1038/nprot.2009.203
